# Bisphenol A Accelerates Toxic Amyloid Formation of Human Islet Amyloid Polypeptide: A Possible Link between Bisphenol A Exposure and Type 2 Diabetes

**DOI:** 10.1371/journal.pone.0054198

**Published:** 2013-01-23

**Authors:** Hao Gong, Xin Zhang, Biao Cheng, Yue Sun, Chuanzhou Li, Ting Li, Ling Zheng, Kun Huang

**Affiliations:** 1 Tongji School of Pharmacy, Huazhong University of Science and Technology, Wuhan, Hubei, People's Republic of China; 2 College of Life Sciences, Wuhan University, Wuhan, Hubei, People's Republic of China; 3 Centre for Biomedicine Research, Wuhan Institute of Biotechnology, Wuhan, Hubei, People's Republic of China; Universidad Miguel Hernández de Elche, Spain

## Abstract

Bisphenol A (BPA) is a chemical compound widely used in manufacturing plastic products. Recent epidemiological studies suggest BPA exposure is positively associated with the incidence of type 2 diabetes mellitus (T2DM), however the mechanisms underlying this link remain unclear. Human islet amyloid polypeptide (hIAPP) is a hormone synthesized and secreted by the pancreatic β-cells. Misfolding of hIAPP into toxic oligomers and mature fibrils can disrupt cell membrane and lead to β-cell death, which is regarded as one of the causative factors of T2DM. To test whether there are any connections between BPA exposure and hIAPP misfolding, we investigated the effects of BPA on hIAPP aggregation using thioflavin-T based fluorescence, transmission electronic microscopy, circular dichroism, dynamic light scattering, size-exclusion chromatography,fluorescence-dye leakage assay in an artificial micelle system and the generation of reactive oxygen species in INS-1 cells. We demonstrated that BPA not only dose-dependently promotes the aggregation of hIAPP and enhances the membrane disruption effects of hIAPP, but also promotes the extent of hIAPP aggregation related oxidative stress. Taken together, our results suggest that BPA exposure increased T2DM risk may involve the exacerbated toxic aggregation of hIAPP.

## Introduction

**Figure 1:**
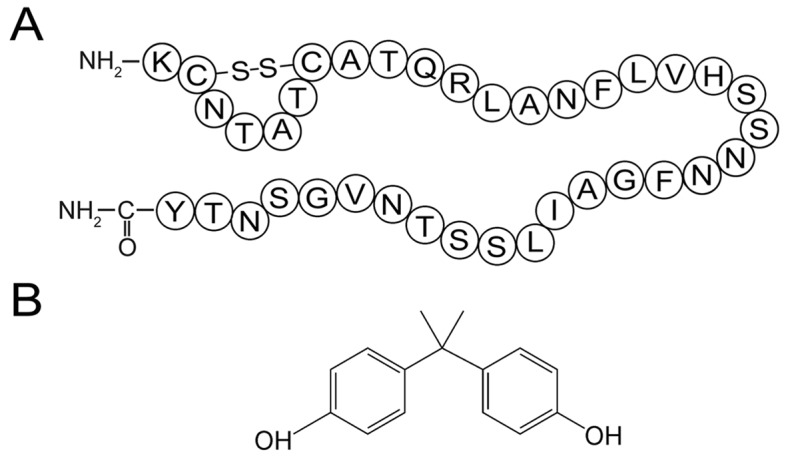
Structures of hIAPP and BPA. (A) Primary sequence of hIAPP with a disulfide bridge between Cys-2 and Cys-7. (B) Chemical structure of bisphenol A.

Diabetes is a panepidemic endocrine disease, with approximately 285 million diagnosed patients worldwide [1]. Non insulin dependent diabetes or type 2 diabetes mellitus (T2DM) accounts for more than 90% of diagnosed diabetes [2]. An important causative factor of T2DM is the misfolding of human islet amyloid polypeptide (hIAPP), which is a 37-residue peptide synthesized and secreted by the pancreatic β-cells (Fig. 1A; [3]). Despite the important physiological functions including glycemic control and regulation of certain hormones [4], hIAPP has a high intrinsic propensity to misfold into toxic oligomers and linear fibrils [5]. During this transition, natively unstructured hIAPP monomers first form β-structure rich oligomers, which further assemble into mature linear fibrils through lateral growth and elongation [6]. The cytotoxicity of hIAPP is generally attributed to the membrane permeabilization ability of hIAPP oligomers and mature fibrils, which cause apoptosis and eventually the onset of diabetes [7]–[10]. Therefore, preventing the formation of toxic hIAPP amyloid has been viewed as a plausible therapeutic approach for T2DM [11].

Bisphenol A (BPA; Fig. 1B) is a compound widely used in polycarbonate, epoxy resins and other polymer materials for manufacturing plastic utensils. The leach of BPA from plastic products is considered an important environmental issue [12]. Humans are exposed to BPA primarily through oral and inhalation routes [13]. BPA exposure is associated with multiple diseases, such as diseases of the reproductive system, nervous system and sexual dysfunction [14]–[16], as well as increased risk of cancer and heart disease [17], [18]. Although the exact molecular mechanisms of BPA toxicity remain unclear, official policies have been enacted or are being considered in many countries to reduce the BPA exposure worldwide [19].

Recent epidemiological evidence suggests that a concentration dependent correlation exists between BPA exposure and the occurrence of diabetes. BPA levels have been found significantly higher in both diagnosed diabetic and borderline diabetic patients than those of non-diabetic subjects [20]. A strong association between high urinary levels of BPA and diabetes has been identified by studying 3400 residents in China that a 37% increase in the incidence of T2DM being observed in subjects with urinary BPA concentration above 1.43 ng/ml compared with the reference concentration (≤0.47 ng/ml) [21]. In a clinic investigation with 1455 adults, the risk of diabetes in the highest BPA concentration group was 2.43 times higher compared with those in the lowest concentration group [22]. Additionally, severe metabolic disorders of glucose homeostasis and insulin resistance, hallmarkers of T2DM that are directly correlated with impaired pancreatic β-cell function, have been also observed in normal mice exposed to BPA [23], [24].

It is well recognized that environmental factors, including multiple metal ions, polyphenols, fatty acids and certain natural products of small molecule size, can affect the toxic misfolding of hIAPP and may cause diabetes [25]–[29]. We thus hypothesize that BPA exposure may associate with diabetes through promoting the toxic aggregation of hIAPP. To test this hypothesis, the effects of BPA on hIAPP aggregation were investigated in this work.

## Materials and Methods

### Materials

Synthetic hIAPP (1–37) was obtained from Genscript Inc. (Piscataway, NJ, USA). Bisphenol A was obtained from Aladdin-reagent (Shanghai, China). Carboxyfluorescein, thioflavin-T (ThT), 2-Oleoyl-1-palmitoyl-sn-glycerol-3-phospho-rac (1-glycerol) sodium salt (POPG) and hexafluoroisopropanol (HFIP) were purchased from Sigma-Aldrich (St. Louis, USA). INS-1 cells were obtained from the China Center for Type Culture Collection (CCTCC). All other chemicals were of the highest grade available.

### hIAPP sample preparation

For all experiments, hIAPP was freshly dissolved in HFIP and vigorously sonicated for 2 min to homogenize the sample. After a short-spin, the solution was diluted to desired concentration in 25 mM sodium phosphate buffer (pH 7.4) containing 50 mM NaCl, and a final HFIP concentration of 1%. Freshly prepared BPA stock solution was then immediately added to desired concentrations,thoroughly mixed and ready for further analysis. The whole preparation process is strictly limited to 5 min.

### Far-UV circular dichroism (CD) and data analysis

CD spectra were obtained with a JASCO-810 spectropolarimeter at 25°C under a constant flow of N_2_. Freshly dissolved hIAPP was diluted to a final concentration of 15 µM,. Spectra were obtained from 260 to 190 nm with a 2 nm bandwidth, 1 s response time, 50 nm/min scanning speed and a 1 mm pathlength. Each sample was measured at least three times and the spectra were averaged to give the final result. Spectra of PBS buffer containing corresponding concentrations of BPA were measured as the baselines. The final spectra were obtained by subtracting corresponding baseline spectrum from sample spectrum, which were further converted to mean residue ellipticity [θ] and were analyzed with the software CDPro using the CONTINLL algorithm as previously described [30].

### Amyloid formation and thioflavin-T (ThT) fluorescence assays

Freshly prepared hIAPP solution (15 µM) was incubated at 25°C for amyloid formation in the presence of different molar ratios of BPA. ThT fluorescence assays were preformed on a Hitachi FL-2700 fluorometer to detect the formation of amyloid at designated time points. The final assay solution contains 25 mM PBS (pH 7.4), 50 mM NaCl and 20 µM thioflavin-T [27]. ThT fluorescence was recorded at 482 nm with an excitation wavelength of 450 nm. PBS buffer containing different concentrations of BPA were measured as the controls. All of the experiments were performed at least three times, and the lag times were calculated as we previously described [31].

### Transmission electronic microscopy (TEM)

The TEM was performed as previously described [32]. Briefly, 5 µl of sample was applied onto a 300-mesh Formvar-carbon coated copper grid. Excess solvent was removed carefully and stained by dropwise addition of 1% freshly prepared uranyl formate followed by air drying. Images were observed under a transmission microscope (Hitachi, Tokyo, Japan) operating at an accelerating voltage of 100 kV.

### Dye leakage assays

POPG was dissolved in chloroform at a concentration of 10 mg/mL. Chloroform was then removed under a stream of N_2_, and samples were dried under vacuum to remove residual chloroform. Multilamellar vesicles were made by mixing dry POPG films with 25 mM PBS (pH 7.4) containing 40 mM carboxyfluorescein. PD-10 columns (Sangon, Shanghai, China) were then used to remove nonencapsulated carboxyfluorescein as previously described [33]. POPG vesicles containing carboxyfluorescein were diluted in 25 mM PBS (pH 7.4) for florescence measurements. hIAPP stock solution was added to POPG vesicles at a final concentration of 1 µM immediately before measurement. The samples were excited at a wavelength of 493 nm, and the emission was detected at 518 nm. The fluorescence signal was recorded for 90 s, POPG vesicles alone were tested as the baseline and the signals of POPG vesicles treated with 0.2% (v/v) Triton X-100 (for complete membrane leakage) were used as the positive control. All measurements were repeated at least three times.

### Size-exclusion chromatography (SEC)

The SEC analysis was performed on a Tosoh TSK GW2000 column (Tokyo, Japan). hIAPP was freshly prepared to a final concentration of 30 µM,mixed with different amounts of BPA, and were immediately injected into a Hitachi L-2000 HPLC system, and the column was eluted with a 20% acetonitrile containing 0.003% TFA at a flow rate of 0.3 ml/min as previously described [34].

### Dynamic light scattering (DLS) analysis

Dynamic light scattering was performed by using a zeta pals potential analyzer (Brookhaven Instruments, New York, USA). 30 µM hIAPP was measured in a 200 µl cuvette incubated at 37°C with a scattering angle of 90°. The starting time for the very first sample scan was marked as time zero. All of the samples were scanned for three times (4 min/scan) and the mean particle size was recorded and analyzed by the multimodal size distribution (MSD) software.

### MTT cell toxicity assay

Pancreatic INS-1 cells were cultured in 1640 medium containing 10% FBS, 1% sodiumpyruvat, 1% penicillin-streptomycin solution and 50 µM β-mecaptoethanol. And cells were plated in 96-well plates at a density of 5×10^4^ cells/well and incubated at 37°C in 5% CO_2_ atmosphere for 24 h. The medium was then replaced with fresh medium containing hIAPP (5 µM) and varied amounts of BPA for 24 h further incubation. Cells treated with BPA or PBS were used as the controls. For MTT assay, cells were co-incubated with 10 µl MTT (5 mg/ml) per well for 4 h. 100 µl formazan buffer was then added to each well and the absorbance was measured at 570 nm [35]. Synergistic effects were analyzed by calculating coefficient of drug interaction (CDI) [36].

### Measurement of reactive oxygen species (ROS)

INS-1 cells were seeded into 6-well plates and treated with or without 10 µM hIAPP and different ratios of BPA for 12 h. The harvested cells were washed by PBS and incubated in 1640 medium containing 10 µM carboxy-H_2_DCFDA (Beyotime, Shanghai, China) for 20 min at 37°C. The cells were then washed twice with PBS and the levels of ROS were detected by a flow cytometer (Beckman, USA) with an excitation wavelength of 485 nm and an emission wavelength of 530 nm [37].

### Statistical analysis

The Kruskal-Wallis test and the Mann-Whitney test were used to evaluate statistical significance. All results were expressed as the mean ± SD. Difference was considered statistically significant at P<0.05.

## Results

### BPA promotes the secondary structure transition of hIAPP

**Figure 2:**
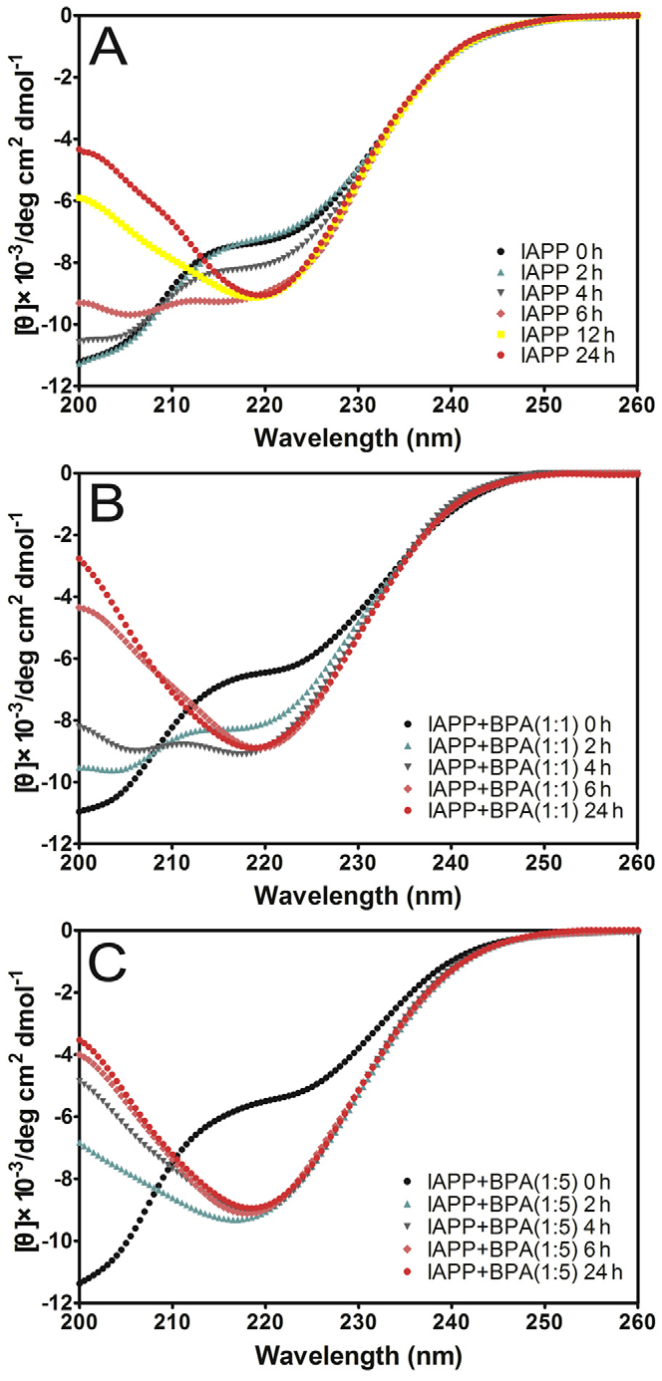
Far-UV circular dichroism spectra. (A) hIAPP; (B) hIAPP with equimolar BPA; (C) hIAPP with 5-fold BPA. hIAPP was dissolved in HFIP first and then diluted to a final concentration of 15 µM. The presence of BPA promoted structural transition to β-rich structures. The spectra were record in the PBS buffer in a timeframe of 24 h.

The transition from random coil structure to predominant β-structure is characteristic of hIAPP amyloid formation [26]. To investigate the effects of BPA on the secondary structure transition of hIAPP during incubation, far-UV CD spectroscopy was applied. At the beginning of incubation, the CD spectrum of hIAPP was characteristic of predominant random coil structure (Fig. 2A), which agreed with previous reports [38], [39]. An intensity increase of a negative band between 210∼220 nm was observed after 6 h of incubation, which represents a conversion from random coil to β-sheet-rich structures (Fig. 2A). After incubating for 12 h, a negative peak near 220 nm was observed, which is a typical sign of β-structures (Fig. 2A). The molar ratio of BPA to hIAPP (1∶1 and 1∶5) tested were determined according to a pilot fluorescence study (data not shown). BPA at molar ratios of 1∶1 and 1∶5 showed no immediate effects on the secondary structure of hIAPP, however, the rate of structural transition was much faster than that of hIAPP alone (Fig. 2B & 2C). In the presence of equimolar amounts of BPA, a notable structural change to the β-sheet conformation was observed at 2 h, which was 4 h earlier than that of hIAPP alone (Fig. 2B). When the molar ratio of BPA was further increased to 5-fold, the broad negative peak at 2 h was similar to the spectrum of hIAPP alone recorded at 12 h with the structural change completion time accelerated to less than 2 h (Fig. 2C). These data suggested BPA dose-dependently promotes the secondary structural transition of hIAPP to the β-sheet-rich structures.

Further CDPro deconvolution analysis confirmed the formation of β-structures (Fig. S2). CD data of hIAPP with or without BPA all showed a significant reduction in random coil structure with an accompanied increase of β-structures (β-sheet and β-turn) after the 24 h incubation (Table S1). For all sample groups, the final percentages of β-structures were greater than 46%. However, the percentage of β-structures at 2 h increased to 35.5% and 43.1% in the presence of 1-fold and 5-fold BPA, respectively, which was significantly higher than that of hIAPP alone (26.5%).

### BPA accelerates the amyloid formation of hIAPP

**Figure 3:**
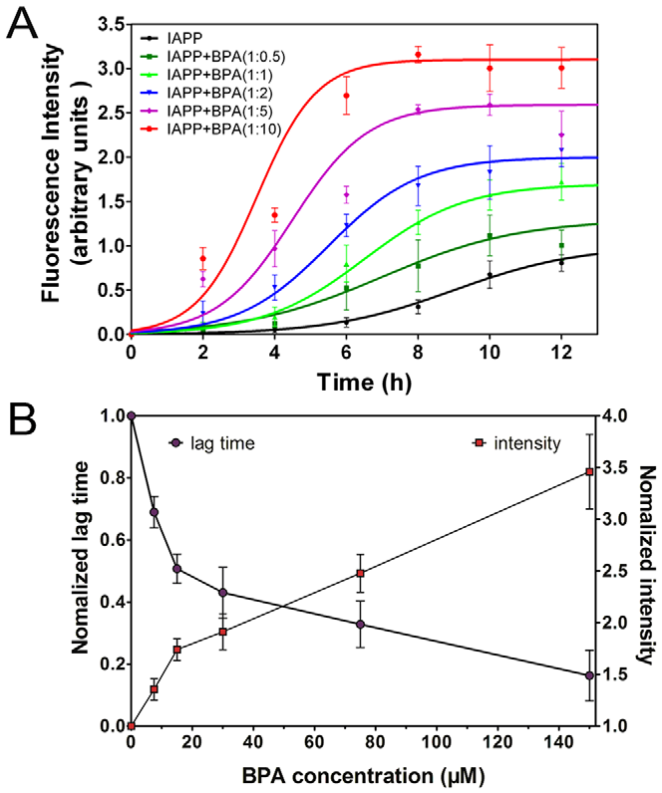
Effects of BPA on hIAPP aggregation. (A) ThT-fluorescence of hIAPP co-incubated with different ratios of BPA recorded in 25 mM PBS (pH 7.4) at 25°C; (B) Normalized lag time (purple circle) and fluorescence intensity (red square). The data of hIAPP without BPA was set as 1. Concentration of hIAPP was 15 µM.

The existence of β-sheet-rich structure is characteristic of amyloid fibrils [40]. Thioflavin-T (ThT) is a fluorescence dye that binds specifically to β-sheet structures and is widely used in probing the emerging β-structures during amyloid formation [41]. hIAPP with different molar ratios of BPA were incubated at 25°C for 24 h. ThT fluorescence was checked every 2 h to monitor the kinetics of fibril formation. hIAPP alone gave a strong ThT emission with a lag time of 5.87±0.77 h (Fig. 3A). The addition of BPA not only dose-dependently shortened the lag time, but also significantly increased the intensity of the maximum fluorescence (Fig. 3B & Table S2). The addition of 0.5-fold BPA shortened the lag time from 5.87±0.77 h to 4.05±0.09 h (P<0.05); when the ratio of BPA/hIAPP was further increased to 1, 2 and 5, the rates of hIAPP misfolding were notably accelerated, with the lag time reduced to 2.98±0.08 h (P<0.05), 2.52±0.23 h (P<0.05) and 1.92±0.19 h (P<0.05), respectively (Table S2). In the presence of 10-fold BPA, the lag time was even reduced to 0.96±0.23 h (P<0.05). The maximum fluorescence intensity also increased notably with the increasing ratios of BPA, with the greatest increment being more than 3 times that of hIAPP alone (Fig. 3B). Control fluorescence spectra recorded with BPA alone indicated BPA by itself does not contribute to this fluorescence intensity increment (Fig. S1).

**Figure 4:**
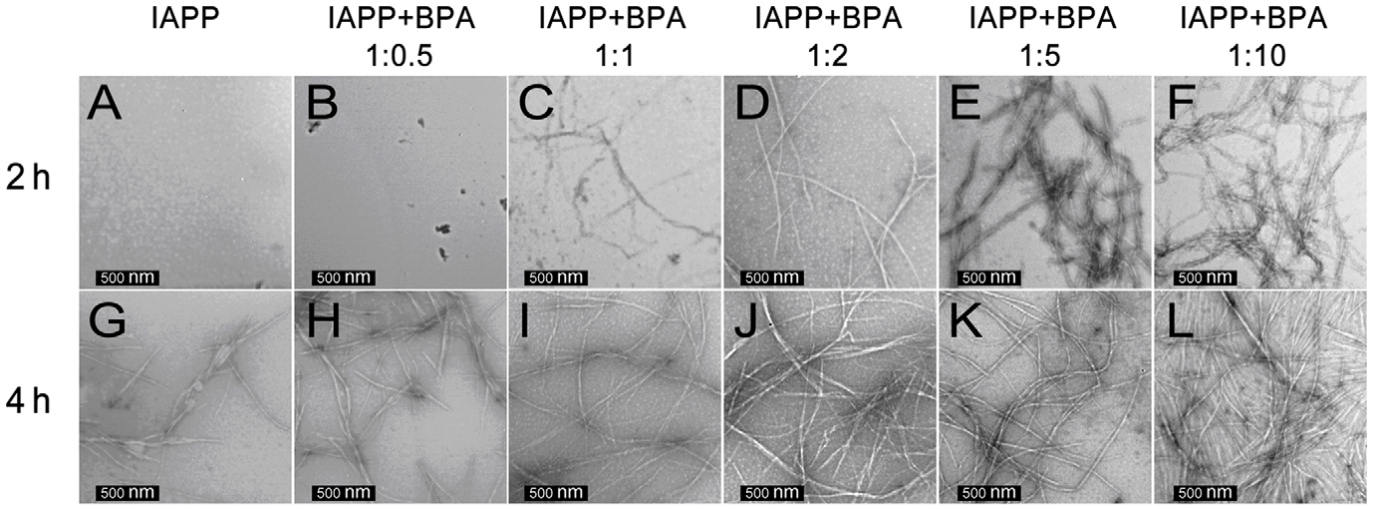
Morphology of hIAPP amyloid formed in the absence or presence of different ratios of BPA as observed by transmission electron microscope. Scale bar represents 500 nm.

Morphology of hIAPP aggregates was observed by TEM. After incubating for 2 h, TEM images of hIAPP with or without 0.5-fold BPA showed no sign of aggregation; scattered short fibrils or protofibrils were found only after 4 h incubation (Fig. 4). Classic long linear hIAPP amyloid fibrils were identified after incubating for 8 h (data not shown). In contrast, typical fibrils appeared much earlier in BPA treated groups (Fig. 4), for example, in the 10-fold BPA group, extensive long linear amyloid fibrils were observed at 2 h, which further grew and formed a fibril mesh at 4 h (Fig. 4). Control TEM images of BPA alone was also obtained and no obvious particle background was found (Fig. S1). These observations are consistent with the results obtained from the ThT fluorescence and CD assays.

### BPA induces the toxic oligomerization of hIAPP

**Figure 5:**
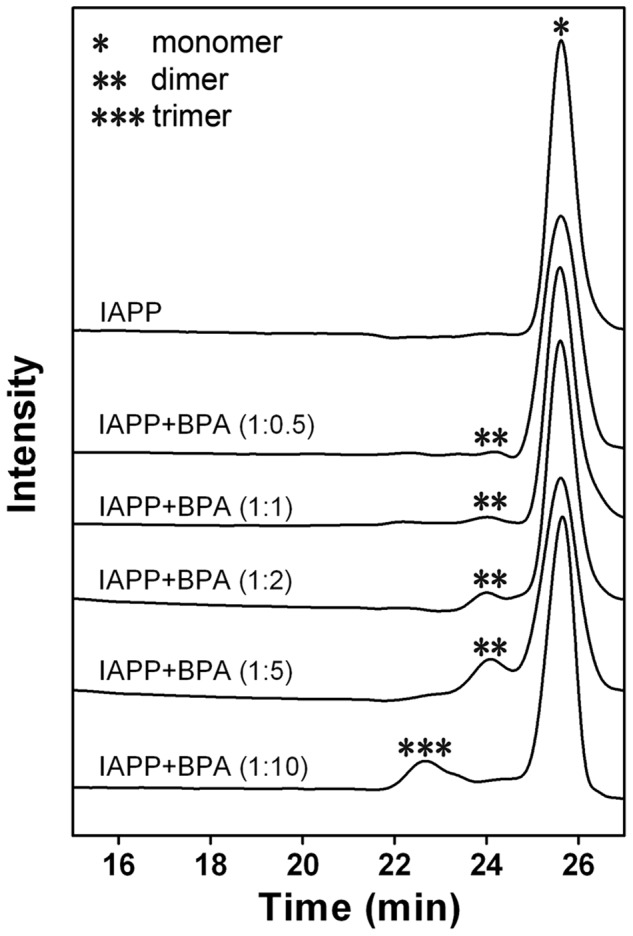
Size-exclusion gel filtration results of hIAPP co-incubated with different molar ratios of BPA. Oligomers of different molecular weights were marked with asterisks.

The oligomerization status of hIAPP was analyzed using size-exclusion chromatography (SEC). Freshly dissolved hIAPP showed a monomer peak eluting at 25 min with a calculated MW of 3.9 kD (Fig. 5). The sample with 5-fold BPA showed a leading peak at 24 min (calculated MW of 7.9 kD), suggesting the formation of an hIAPP dimer. In the presence of 10-fold BPA, the dimer peak almost disappeared with a new peak eluting at 22.5 min (calculated MW of 12 kD), indicating the formation of a trimer. These data suggested BPA dose-dependently accelerates the oligomerization of hIAPP within a short time span.

**Figure 6:**
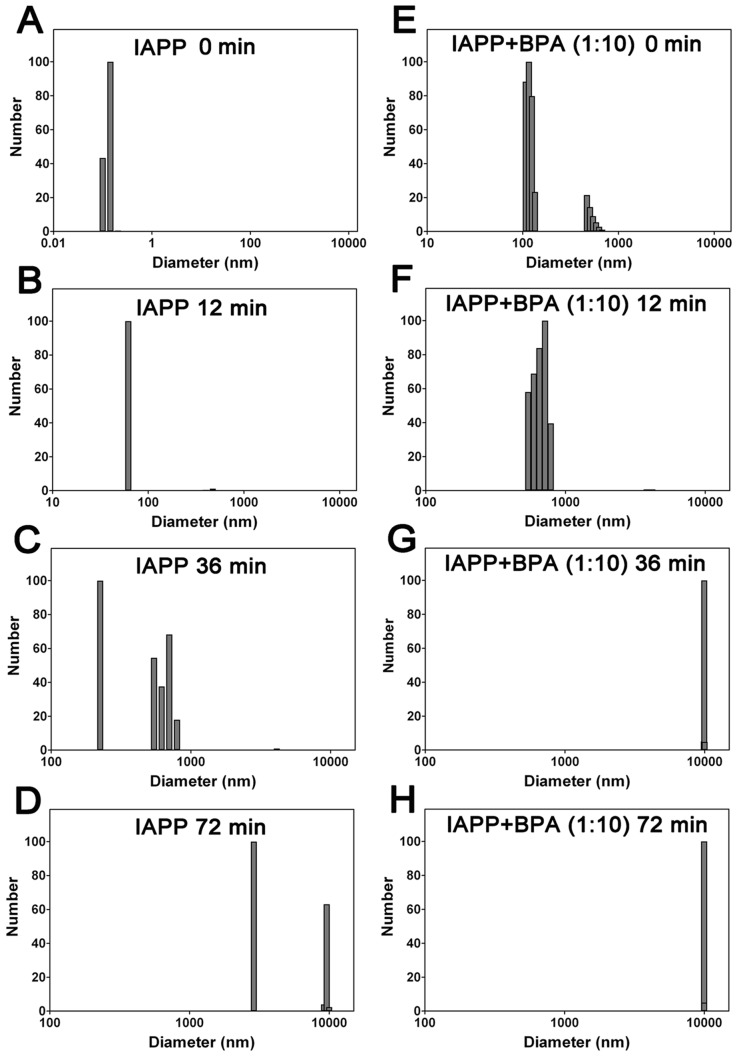
Dynamic light scattering detected hIAPP particle size distributions. (A–D) hIAPP and (E–H) hIAPP co-incubated with 10-fold BPA. Concentration of hIAPP was 30 µM. The samples were incubated and measured at 37°C. The presence of BPA increased the number of large particles, which represent oligomers and fibrils. The starting time for the first scan is defined as time zero.

Dynamic light scattering (DLS) measurements were then performed to monitor particle size distributions during the aggregation of hIAPP. Gradually increased particle sizes were observed during incubation. The average diameter of native hIAPP monomers was 0.1 nm (Fig. 6A), the hydrodynamic diameter increased to 62 nm after incubating for 12 min, suggesting the formation of large size oligomers (Fig. 6B). 36 min later, protofibrils were observed with an average particle size of 525 nm, which further assembled into mature fibrils with an average particle size of 2890 nm at 72 min (Fig. 6C & 6D). This process was significantly accelerated in the presence of 10-fold BPA, the average particle size measured from the first scan was 205 nm (Fig. 6E), significantly higher compared to the companying BPA-free hIAPP sample (0.1 nm), suggested a rapid oligomerization; protofibrils with an average size of 541 nm were observed at 12 min (Fig. 6F) and fibrils with sizes greater than 5000 nm appeared after only 24 min (data not shown). The average particle size reached 10000 nm at 36 min and no more significant change was observed with further incubation (Fig. 6G & 6H). Control studies (containing just BPA as the blank) suggested no interference (signals) from BPA.

### BPA increases the INS-1 cells apoptosis caused by the hIAPP

**Figure 7:**
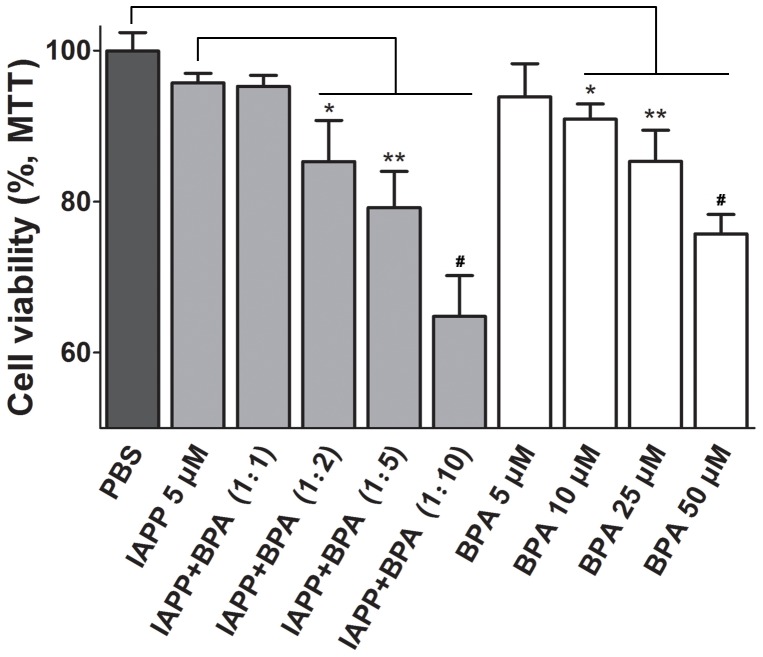
INS-1 cell viabilities in the presence of BPA as determined by the MTT assay. Untreated INS-1 cells treated with equal volume of PBS was used as the negative control.*, P<0.05; **, P<0.01; and #, P<0.001.

The cytotoxic process of hIAPP fibrillation has been shown to decrease the viability of pancreatic INS-1 cells [42]. We found INS-1 cells co-incubated with 5 µM hIAPP for 24 h resulted in a reduced cell viability of 95.9±0.8% (P<0.05) compared with the untreated control cells. And the cytotoxicity was dose-dependently enhanced in the presence of BPA (Fig. 7). Compared with cells treated with hIAPP alone, the cell viability dropped to 85.3±3.2% (P<0.01) at 2 fold molar amount of BPA in combination with hIAPP; whereas at ratios of 5 and 10, it further dropped to 79.2±4.8% (P<0.01) and 62.1±5.3% (P<0.001). No obvious effect was found at BPA ratio of 1∶1 (Fig. 7) or lower (data not shown). Since BPA by itself also showed strong cytotoxicity,CDI was calculated to analyze whether BPA and hIAPP have synergic action on the cytotoxicity. Except the equimolar group, CDIs of the three BPA groups at higher ratio are all below 1, suggesting the existence of a synergistic effect (Table S3). These data indicate BPA may have promotional effect on hIAPP cytotoxicity in certain concentration range.

### BPA increases the membrane disruption ability of hIAPP

**Figure 8:**
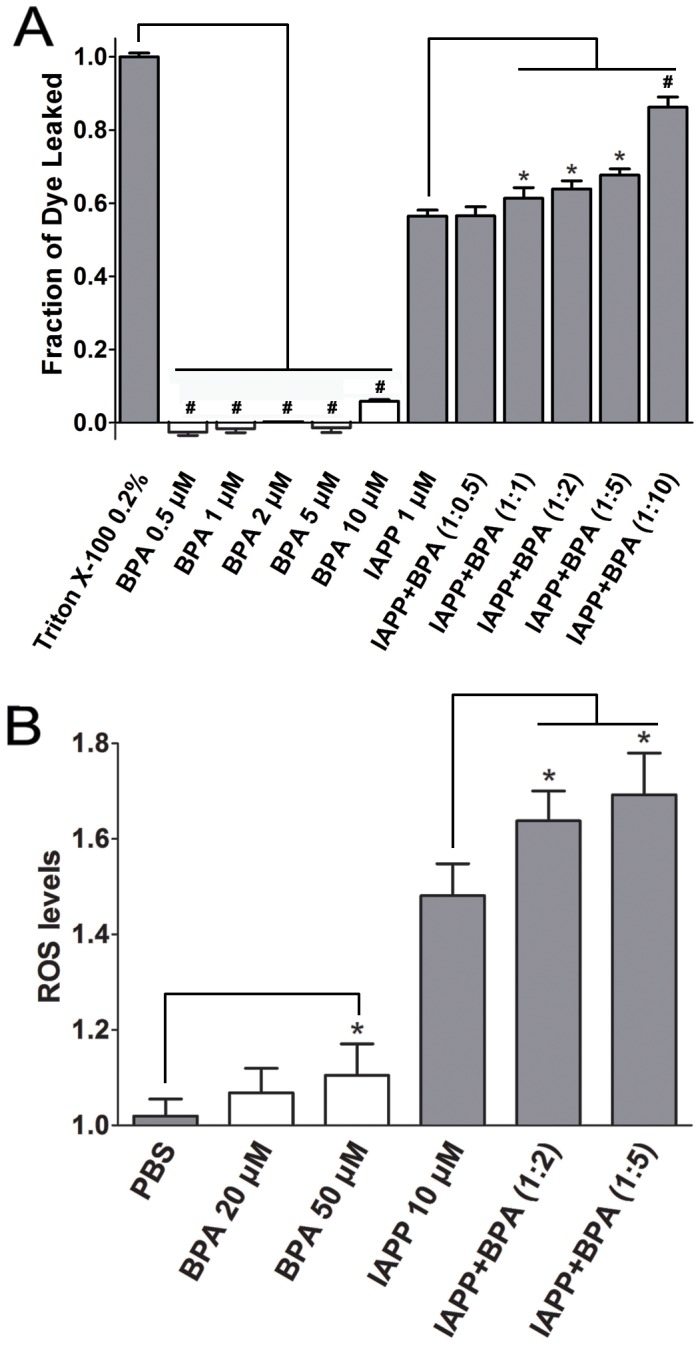
Dye leakage and ROS assays of hIAPP co-incubated with BPA. (A) Fluorescence dye leakage levels of hIAPP in the presence of different ratios of BPA. 0.2% Triton X-100 treated carboxyfluorescein-containing POPG micelles were used as the positive control. . (B) ROS levels of INS-1 cell treated with hIAPP and BPA. Untreated INS-1 cells co-incubated with equal volume of PBS was used as negative control. Concentrations of hIAPP used for dye leakage assay and ROS assay were 1 µM and 10 µM, respectively. *, P<0.05.

hIAPP oligomers and mature fibrils have been reported to disrupt β-cell membranes and release intracellular contents, which results in β-cell apoptosis and eventually diabetes [9]. We performed dye leakage assays that use artificial micelles containing fluorescence dye to probe the membrane disruption capacity of hIAPP. The control study suggested BPA alone did not disrupt membranes in the range of 0.5–5 µM, and slight membrane disruption (<6%) was observed in the presence of 10 µM BPA. In contrast, 1 µM hIAPP disrupted membrane by 56.5±0.1% within 90 s (Fig. 8A), which is consistent with a previous report [43]. The presence of BPA in ratios from 1 to 10 all substantially increased the membrane disruption of hIAPP compared to that of the hIAPP alone. In the presence of 2-fold and 5-fold BPA, the membrane disruption was increased to 64.7±0.2% (P<0.05) and 68.9±0.1% (P<0.05), respectively. The 10-fold BPA showed the highest penetration ability of 86.3±0.1% (P<0.001; Fig. 8A).

### BPA elevates levels of reactive oxygen species (ROS)

It has been well documented that amyloid formation is also associated with the generation of toxic ROS [44]. To test whether BPA exposure has any effect on the ROS generation, which is associated with amyloid formation of hIAPP, the ROS levels were measured. Since it has been shown that high concentrations of BPA may directly promote the production of ROS of cells [45], we first treated INS-1 cells with increased concentrations of BPA. Only slightly increased ROS levels was observed at BPA concentrations below 50 µM (data not shown), so 20 µM and 50 µM BPA were used in the following experiments. Compared with untreated INS-1 cells, 10 µM hIAPP caused a 46.2% increase in the ROS level (P<0.05), which agreed with a previous report [46]. For INS-1 cells treated with both hIAPP (10 µM) and BPA (20 µM or 50 µM), the ROS levels raised another 15.6% and 21.1% compared to the cells treated only with hIAPP (P<0.05; Fig. 8B).

## Discussion

hIAPP has a strong tendency to form toxic oligomers and fibrils that lead to pancreatic β-cell apoptosis and eventually the onset of T2DM [47]. It is clear from our results that BPA promotes hIAPP aggregation in a dose-dependent manner, which is supported by the significantly accelerated aggregation lag time as well as the enhanced fluorescence intensity that reflects the orderly β-structures formed (Fig. 3B). CD data further confirms the accelerated transition of hIAPP from unordered structure to β-structure in the presence of BPA (Fig. S2). The helical intermediates are thought to play a role in hIAPP aggregation [48], [49], the transition from helix to β-sheet structure may cause the reduction of helical structures as what we observed (Fig. S2).

It is well established that the toxic hIAPP oligomers can disrupt the islet β-cell membrane and lead to permeabilization. In MTT study, we observed the cytotoxicity of hIAPP on INS-1 cells rose sharply with the addition of BPA (Fig. 7). The observed strong cytotoxicity by BPA alone also agree with previous studies which suggested BPA itself also disrupt the cell function through stimulating the estrogen-receptor and several other apoptosis-related pathways [50]–[53]. It is interesting to note that the increased toxic effect of hIAPP in combination with BPA was only observed at high BPA to hIAPP ratios but not at the lower ratios. The CDI was calculated to explore the potential BPA and hIAPP interaction, and it was found that the two compounds showed a synergistic exacerbation of cototoxicity especially at high molar ratios of BPA.

Further analysis was conducted to distinguish the direct molecular toxic effects of BPA on live cells from its interaction with hIAPP. Dye leakage assays were performed to monitor exclusively the membrane disruption property of hIAPP in the presence of BPA. The hIAPP oligomers have been proven to bind and penetrate membranes more efficiently than monomers and are regarded as an important causative factor to β-cell death [47]. Serious dye leakage was observed in the hIAPP group and was dose-dependently enhanced in the presence of BPA (Fig. 8). It is interesting to note that no evident hIAPP aggregation was identified within a short incubation time at a low concentration (1 µM) as suggested by the ThT and CD results (data not shown), whereas hIAPP at this concentration immediately caused significant membrane disruption in dye leakage assays. This may be explained by a recent report that hIAPP form oligomers much faster in the presence of membrane structures [54]. These data suggested that BPA significantly increases the ability of hIAPP to disrupt membranes.

**Figure 9:**
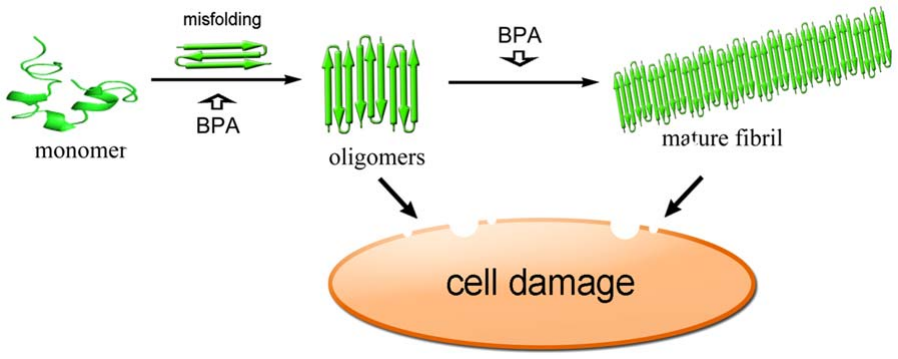
A schematic representation of hIAPP aggregation pathway. BPA may promote the oligomerization of hIAPP and form pores on membrane which disrupt the membrane and cause the leakage of cellular contents, moreover, permeabilization of lipid bilayers may cause the elevation of intracellular calcium levels and lead to the generation of ROS.

Oxidative stress induced cytotoxicity was another mechanism underlying amyloid-related β-cell apoptosis besides direct membrane disruption. Amyloid formation has been reported to associate with ROS generation [46]. hIAPP oligomers may form pores on membrane and lead to permeabilization of lipid bilayers [55]. The generalized increase in membrane permeability results in intracellular calcium elevation which disrupts mitochondrial function and finally increases ROS generation [56], [57]. In this study, ROS accumulation is also observed in INS-1 cells treated with hIAPP. The ROS levels rose significantly in the presence of BPA and hIAPP, while BPA by itself has little effect on ROS levels (Fig. 8B), suggesting BPA has a synergistic effect on the ROS production related to hIAPP amyloid formation. A summary of possible molecular scheme is provided for the toxic effects of BPA on the formation of hIAPP amyloid which further result in cell damage (Fig. 9).

The influence on human health of BPA exposure is regarded as an accumulative process because of its widespread penetration in daily life [58]. The BPA tolerable intake has been set as 50 µg/kg/day [58], but adverse effects at lower BPA concentrations in animal studies have been demonstrated, which may lead to the requirement of a new risk assessment for BPA [59]. BPA concentrations in human blood (serum and plasma) are in the range of 0.3–4.4 ng/ml (1.3–19.4 nM) in developed countries [13]. In contrast, physiological circulating concentrations of hIAPP are below 10 pM in fasted non-diabetic people and rise up to over 20 pM after a meal [60], suggesting the physiological ratio of BPA to hIAPP may actually be much higher than those used in the present study. Moreover, since BPA exposure is a continuous and accumulative process, it is logical to expect long-term BPA exposure may be accompanied with accelerated hIAPP amyloid formation and β-cell apoptosis, and eventually a higher risk of T2DM. In a recent report, BPA at near physiological concentration also showed direct toxicity [61]. Due to the detection sensitivity limitation of existing biophysical technologies, in the present study, we tested the interaction between hIAPP and BPA at much higher concentrations *in vitro*. Therefore the system we used may be considered as a model that simulates physiological interactions at accelerating rates. Future biophysical study with novel experimental methods which can tackle hIAPP and BPA interaction at physiological conditions will be important.

In summary, our data provide the evidence that BPA exposure concentration-dependently accelerates the toxic amyloid formation, exacerbates the toxic membrane disruption of hIAPP and promotes the levels of toxic ROS generated by hIAPP in vitro. Our study suggest that in addition to direct biological effect, long-term BPA exposure may also have adverse effects on hIAPP amyloid formation that eventually contribute to the onset of T2DM. The results may provide a new angle on how BPA exposure influences the risk of diabetes from hIAPP aggregation related pathogenesis. Moreover, since BPA also possess other important biological effects including estrogen-like function, it will be interesting to explore the effect of BPA exposure on physiological functions of hIAPP, for example, hIAPP secretion and hIAPP related insulin resistance. In addition to hIAPP, a great variety of amyloidogenic proteins such as amyloid β peptide and α-synuclein, are known to form extracellular amyloid deposits that induce human diseases including Alzheimer's disease and Parkinson's disease [62]. It will be of future interest to study how BPA exposure may affect the misfolding of those amyloidogenic proteins.

## Supporting Information

Table S1 Secondary structure compositions (in %) of hIAPP incubated with different ratios of BPA. The spectra were calculated with the algorithm CONTINLL provided by the CDPro package using SDP42 as the reference set.
(DOC)

Table S2 Amyloidogenic properties of hIAPP incubated with different ratios of BPA.
(DOC)

Table S3 Synergistic effects of BPA and hIAPP.
(DOC)

Figure S1 Aggregation analysis of hIAPP co-incubated with BPA and corresponding BPA control. (A) ThT-fluorescence profile over 24 h incubation. hIAPP concentration was 15 µM. (B) Size-exclusion gel filtration profile. (C) TEM images of samples incubated for 4 h.
(DOC)

Figure S2 Percentages of secondary structure contents of hIAPP in the presence of different molar ratios of BPA as calculated by the CONTINLL algorithm. (A) hIAPP; (B) hIAPP with equal BPA; (C) hIAPP with 5-fold molar BPA.
(DOC)
