# Supplementary material for: Bisphenol A Accelerates Toxic Amyloid Formation of Human Islet Amyloid Polypeptide: A Possible Link between Bisphenol A Exposure and Type 2 Diabetes
Source: PLoS One. 2013 Jan 23;8(1):e54198. doi: 10.1371/journal.pone.0054198 (PMC3553173; doi:10.1371/journal.pone.0054198)
Supplement: Table S1 — Secondary structure compositions (in %) of hIAPP incubated with different ratios of BPA. The spectra were calculated with the algorithm CONTINLL provided by the CDPro package using SDP42 as the reference set. (DOC) [file pone.0054198.s001.doc]

***Table S1.*** Secondary structure compositions (in %) of hIAPP incubated with different ratios of BPA. The spectra were calculated with the algorithm CONTINLL provided by the CDPro package using SDP42 as the reference set.

| **Samples** | α-helix | β-structure  （β-sheet + β-turn） | unordered |
| --- | --- | --- | --- |
| hIAPP |  |  |  |
| 0 h | 16.4 | 24.7 （11.5+13.2） | 58.9 |
| 2 h | 16.2 | 26.5 （12.5+14.0） | 57.2 |
| 4 h | 16.6 | 27.7 （12.7+15.0） | 55.8 |
| 6 h | 16.0 | 33.7 （16.0+17.7） | 50.4 |
| 12 h | 10.3 | 41.8 （21.3+20.5） | 48.0 |
| 24 h | 7.8 | 46.4 （24.6+21.8） | 45.8 |
| hIAPP : BPA (1:1) |  |  |  |
| 0 h | 16.4 | 24.4 （11.3+13.1） | 59.2 |
| 2 h | 11.7 | 35.3 （17.9+17.4） | 53.0 |
| 4 h | 11.6 | 39.1 （19.9+19.2） | 49.3 |
| 6 h | 9.2 | 44.2 （23.1+21.1） | 46.6 |
| 24 h | 10.2 | 46.9 （24.8+22.1） | 42.8 |
| hIAPP :BPA (1:5) |  |  |  |
| 0 h | 9.8 | 34.7 （20.7+14.0） | 55.5 |
| 2 h | 9.8 | 43.1 （21.9+21.2） | 47.0 |
| 4 h | 9.9 | 44.8 （23.5+21.3） | 45.4 |
| 6 h | 8.3 | 48.3 （25.7+22.6） | 43.5 |
| 24 h | 9.4 | 46.8 （24.9+21.9） | 43.8 |
