# Supplementary material for: Bisphenol A Accelerates Toxic Amyloid Formation of Human Islet Amyloid Polypeptide: A Possible Link between Bisphenol A Exposure and Type 2 Diabetes
Source: PLoS One. 2013 Jan 23;8(1):e54198. doi: 10.1371/journal.pone.0054198 (PMC3553173; doi:10.1371/journal.pone.0054198)
Supplement: Table S2 — Amyloidogenic properties of hIAPP incubated with different ratios of BPA. (DOC) [file pone.0054198.s002.doc]

***Table S2.*** Amyloidogenic properties of hIAPP incubated with different ratios of BPA. ^a^

| **Samples** | Fibril formation ^b^ | | |
| --- | --- | --- | --- |
|  | lag time (hr) | t_50_ (hr) | intensity ^c^ |
| hIAPP | 5.87 ± 0.77 | 9.3 ± 0.3 | 1.00 ± 0.04 |
| hIAPP : BPA （1:0.5） | 4.05 ± 0.09** | 8.0 ± 1.0 | 1.36 ± 0.01* |
| hIAPP : BPA （1:1） | 2.98 ± 0.07** | 7.0 ± 1.7 | 1.74 ± 0.01* |
| hIAPP : BPA （1:2） | 2.53 ± 0.23** | 6.0 ± 1.7 | 1.90 ± 0.03* |
| hIAPP : BPA （1:5） | 1.93 ± 0.19** | 5.3 ± 2.3 | 2.44 ± 0.11** |
| hIAPP : BPA （1:10） | 0.96 ± 0.23# | 5.0 ± 1.0 | 3.46 ± 0.13** |

^a^ All assays were repeated at least three times.

^b^ The concentration of hIAPP was 15 μm.

^c^ The fluorescence intensity of hIAPP alone was set as 1.

*, P < 0.05; **, P < 0.01 and #, P < 0.001 compared with the control hIAPP (15 μM).
