# Supplementary material for: Bisphenol A Accelerates Toxic Amyloid Formation of Human Islet Amyloid Polypeptide: A Possible Link between Bisphenol A Exposure and Type 2 Diabetes
Source: PLoS One. 2013 Jan 23;8(1):e54198. doi: 10.1371/journal.pone.0054198 (PMC3553173; doi:10.1371/journal.pone.0054198)
Supplement: Table S3 — Synergistic effects of BPA and hIAPP. (DOC) [file pone.0054198.s003.doc]

***Table S3.*** Synergistic effects of BPA and hIAPP.

| **Samples** | hIAPP(5 μM) | | | |
| --- | --- | --- | --- | --- |
|  | BPA  (5 μM) | BPA  (10 μM) | BPA  (25 μM) | BPA  (50 μM) |
| CDI | 1.058 | 0.978 | 0.967 | 0.855 |

Coefficient of drug interaction (CDI) was calculated with the equation CDI = AB/(A×B) （AB，relative cell viability of the combination; A or B，relative cell viability of the single agent groups）. CDI﹤1 indicates a synergistic effect; CDI=1 indicates an additive effect; CDI﹥1 indicates an antagonistic effect.
