# Supplementary material for: Bisphenol A Accelerates Toxic Amyloid Formation of Human Islet Amyloid Polypeptide: A Possible Link between Bisphenol A Exposure and Type 2 Diabetes
Source: PLoS One. 2013 Jan 23;8(1):e54198. doi: 10.1371/journal.pone.0054198 (PMC3553173; doi:10.1371/journal.pone.0054198)
Supplement: Figure S1 — Aggregation analysis of hIAPP co-incubated with BPA and corresponding BPA control. (A) ThT-fluorescence profile over 24 h incubation. hIAPP concentration was 15 µM. (B) Size-exclusion gel filtration profile. (C) TEM images of samples incubated for 4 h. (DOC) [file pone.0054198.s004.doc]

***
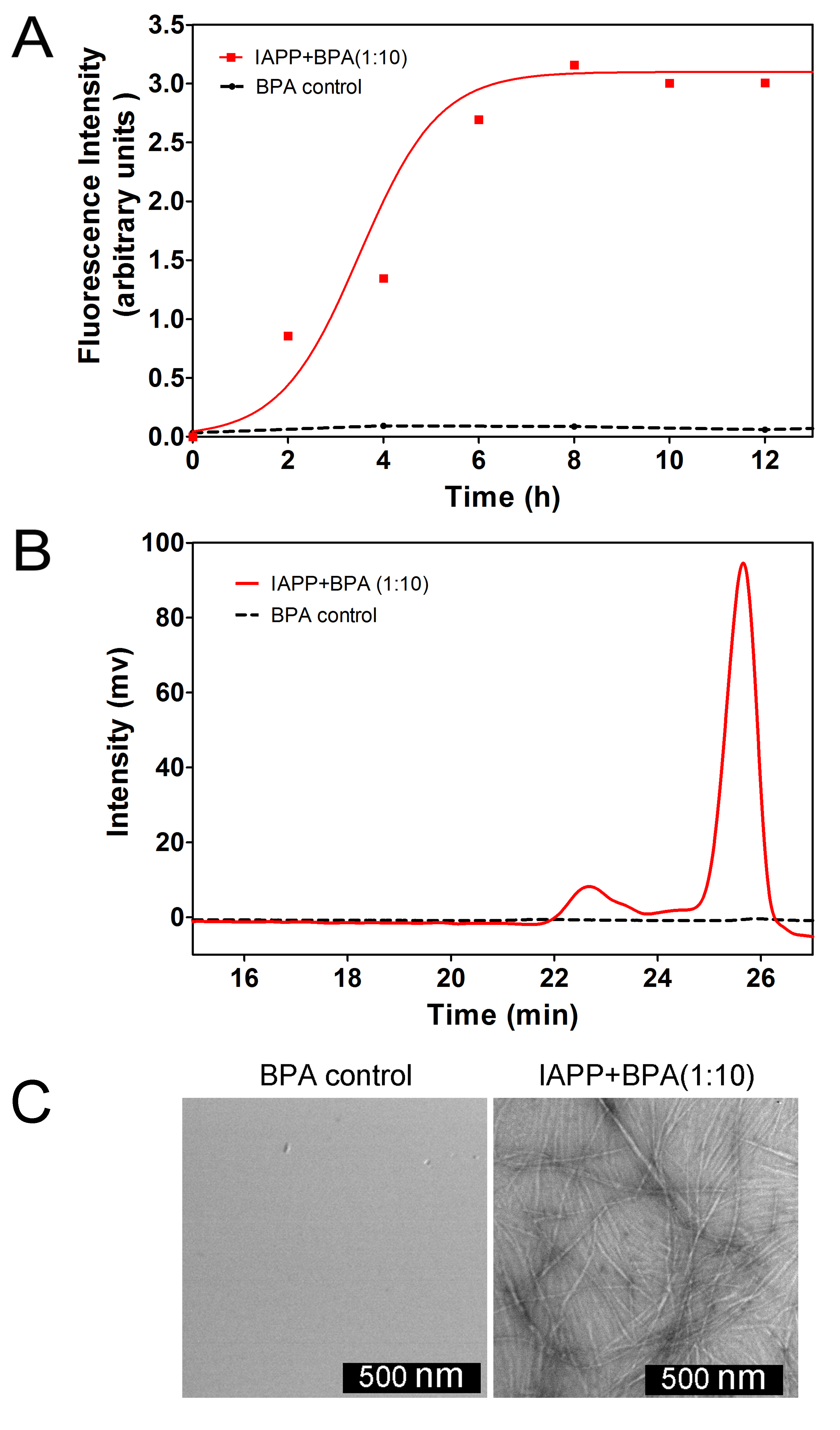
***

***Figure S1.*** Aggregation analysis of hIAPP co-incubated with BPA and corresponding BPA control. (A) ThT-fluorescence profile over 24 h incubation. hIAPP concentration was 15 μM. (B) Size-exclusion gel filtration profile. (C) TEM images of samples incubated for 4 h.
