# Supplementary material for: Bisphenol A Accelerates Toxic Amyloid Formation of Human Islet Amyloid Polypeptide: A Possible Link between Bisphenol A Exposure and Type 2 Diabetes
Source: PLoS One. 2013 Jan 23;8(1):e54198. doi: 10.1371/journal.pone.0054198 (PMC3553173; doi:10.1371/journal.pone.0054198)
Supplement: Figure S2 — Percentages of secondary structure contents of hIAPP in the presence of different molar ratios of BPA as calculated by the CONTINLL algorithm. (A) hIAPP; (B) hIAPP with equal BPA; (C) hIAPP with 5-fold molar BPA. (DOC) [file pone.0054198.s005.doc]

***
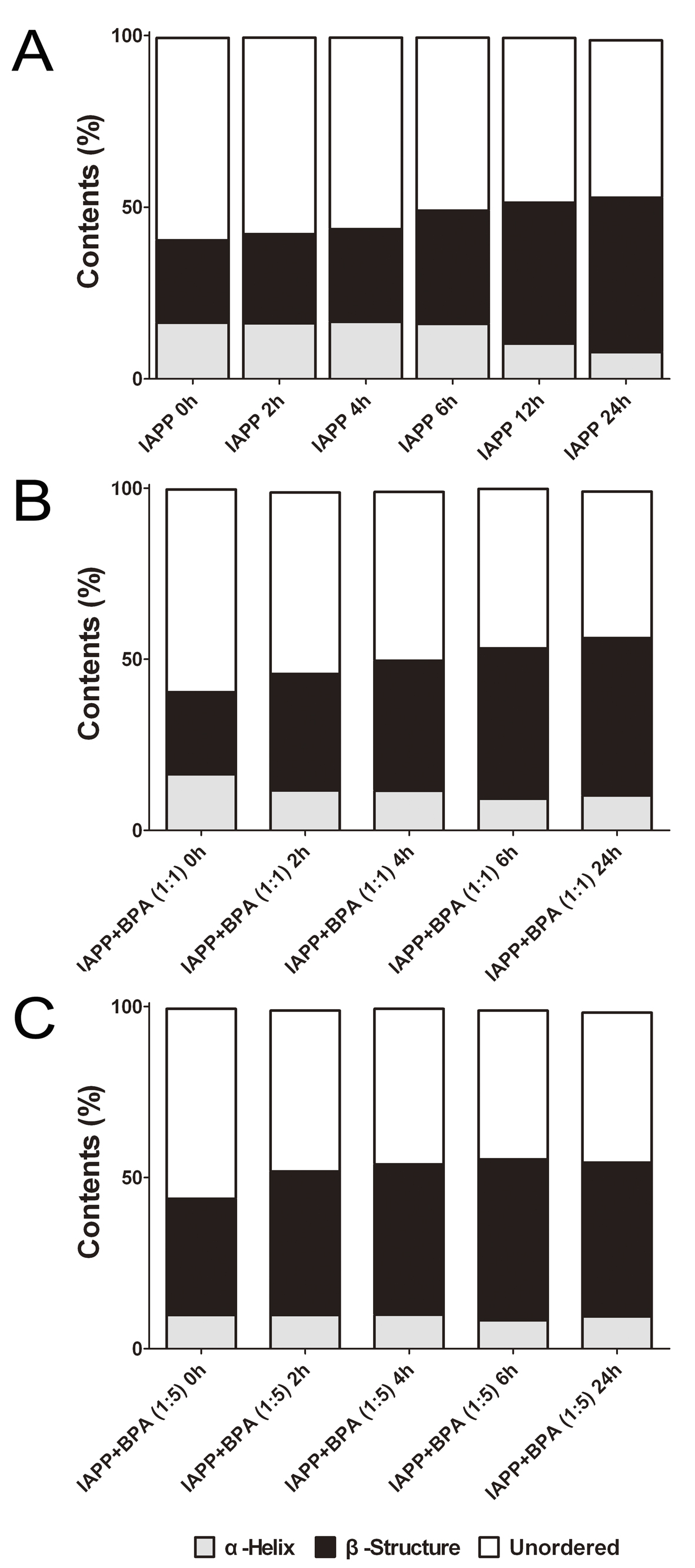
***

***Figure S2.*** Percentages of secondary structure contents of hIAPP in the presence of different molar ratios of BPA as calculated by the CONTINLL algorithm. (A) hIAPP; (B) hIAPP with equal BPA; (C) hIAPP with 5-fold molar BPA.
